# Supplementary material for: Effect of optical correction by fully corrected glasses on postural stability
Source: PLoS One. 2020 Jul 10;15(7):e0235919. doi: 10.1371/journal.pone.0235919 (PMC7351164; doi:10.1371/journal.pone.0235919)
Supplement: Attachment — Submitted filename: review.docx [file pone.0235919.s002.docx]

1. Line 21: New glasses can be interpreted as either first-time wearing glasses, or as updated prescription. This issue is repeated throughout the manuscript.
2. Introduction appears to be well-research and strongly motivated.
3. Line 87: What is “real” myopia?
4. **Subjects**
   There seems to be a significant confusion here in terms of experimental design.

The Myopia and Hyperopia groups are receiving two different conditions.

a. Hyperopia is receiving glasses for the first time: a novel experience
b. Myopia is receiving updates lenses: a non-novel experience

While any significant change in prescription will have an adjustment period, it is important to investigate the postural differences between an updated prescription and the entirely new experience of getting glasses. Until there is data stating that the differences between these two conditions are statistically non-significant, you cannot proceed on the assumption that they are comparable. That is to say, the outcomes of these two groups cannot be compared, because they are essentially receiving different treatments (first time glasses vs updated lenses).

I do commend the researchers for ensuring that the average diopter change to the lenses was comparable between the myopia and hyperopia group. With this being stated, in order to publish the data in this study, these two groups should be separated into either two separate papers or into two distinctively different sections of the paper.

c. Effects of updated prescriptions in patients with Myopia on postural sway.
d. Effects of first-time glasses worn by patients with Hyperopia on postural sway.

5. Line 119: “posture”
6. Line 168: A paired t-test is appropriate for two sets of observations from a same subject. This is done throughout the manuscript, but a two-way ANOVA is most appropriate for the conclusions being drawn here. However, this is still not sufficient, because of the two different starting conditions.

**Discussion**:

7. Line 250: “Man” should be “Many”

While this manuscript is well-motivated, the immediate issue of the experimental design is glaring. There may be significant differences between myopia and hyperopia when receiving new glasses, as well as when receiving an updated prescription. There is not enough collected data to properly complete either of those comparisons.

Ideally, a repeated measures ANOVA would be the best statistical test for your end goal. (Myopia x Hyperopia) x (Without x Old x New) While the data in this manuscript may be usable, the emphasis between these two very different starting conditions needs to be stressed.

At this point, major revision is advisable at a minimum. The manuscript will need to be divided into two sections, or redone as two separate papers.
