# Supplementary material for: Effect of optical correction by fully corrected glasses on postural stability
Source: PLoS One. 2020 Jul 10;15(7):e0235919. doi: 10.1371/journal.pone.0235919 (PMC7351164; doi:10.1371/journal.pone.0235919)
Supplement: Attachment — Submitted filename: Respose to Reviewer (Minor revision).docx [file pone.0235919.s004.docx]

Manuscript number: PONE-D-20-06787R1

Title: Effect of optical correction by fully corrected glasses on postural stability

**Editor Comments**

Dear Dr. Kim,
Thank you for submitting your manuscript to PLOS ONE. After careful consideration, we feel that it has merit but does not fully meet PLOS ONE’s publication criteria as it currently stands. Therefore, we invite you to submit a revised version of the manuscript that addresses the points raised during the review process.
As you will see, both Reviewers approved your revisions, and request only a few modest changes. I endorse their recommendations. I look forward to accepting a modestly revised final version.
Please submit your revised manuscript by Jul 26 2020 11:59PM. If you will need more time than this to complete your revisions, please reply to this message or contact the journal office at plosone@plos.org. When you're ready to submit your revision, log on to <https://www.editorialmanager.com/pone/> and select the 'Submissions Needing Revision' folder to locate your manuscript file.

**Response of the authors:** We are so grateful to the editor for reviewing our manuscript PONE-D-20-06787R1, titled “Effect of optical correction by fully corrected glasses on postural stability,” resubmitted for publication in PLOS ONE. We appreciate the reviewers and your positive evaluations. We revised the manuscript based on a point-by-point response to the reviewers’ comments. Please find the revised manuscript with the changes highlighted in colored text.

**Review Comments to the Author**

Please use the space provided to explain your answers to the questions above. You may also include additional comments for the author, including concerns about dual publication, research ethics, or publication ethics. (Please upload your review as an attachment if it exceeds 20,000 characters)

**※ Reviewer #1 (Marked as R1):**

**R1-1**. Line 198: Add clarity here, by stating which analysis was used. Repeated measures ANOVA, due to the presence of F. Same should go for any analysis (t-test, post-hoc, ANOVAs, etc).

Response to [R1-1]: Thank you for your detailed comments. As the reviewer pointed out, each statistical method was added to the Results section as follows. (Please see the lines in the “Revised Manuscript with Track Changes”)

Lines 203-204: “(F = 4.561, *p* < 0.05 by repeated measures ANOVA)”

Lines 206-208: “(*p* < 0.05 for no glasses vs. old glasses, *p* < 0.05 for no glasses vs. updated glasses by LSD post hoc analysis)”

Line 210: “(*p* > 0.05 for old glasses vs. updated glasses by LSD post hoc analysis)”

Lines 214-215: “(t = 1.006, *p* > 0.05 by paired t-test)”

Lines 230-231: “(F = 4.724, *p* < 0.05 by repeated measures ANOVA)”

Lines 234-235: “(*p* < 0.05 for no glasses vs. old glasses, *p* < 0.05 for no glasses vs. updated glasses by LSD post hoc analysis)”

Line 237-238: “(*p* > 0.05 for old glasses vs. updated glasses by LSD post hoc analysis)”

For further clarification, footnotes were added to Figures 3 and 4 as follows.

The footnote in Fig 3 (Please see lines 219-221): “^*^*p* < 0.05: significantly different depending on each visual condition according to LSD (least significant difference) post hoc analysis by repeated measures ANOVA”

Error bars indicate the standard error (SE) of the mean.

The footnote in Fig 4 (Please see line 225): “Error bars indicate the standard error (SE) of the mean”

**R1-2**. Line 209: “instability index was more significantly decreased”
You do state afterwards that this is not statistically significant. I would remove the “significantly” in line 209 to bolster clarity.

Response to [R1-2]: Thank you for your comments. I agree with the reviewers. “Significantly” was removed, as you suggested (Please see line 213).

**R1-3**. Review indenting/consistency in formatting, overall. I greatly appreciate your response, and wish you the best for future research along this path.

Response to [R1-3]: Thank you for your comments. We revised the format of the main body (Paragraph

Indentation, References et.al). We are very grateful to the reviewer for reviewing our manuscript.

**※ Reviewer #2 (Marked as R2):**

This manuscript is much improved - thank you for addressing previous concerns.
there are a couple of minor comments that should be addressed:

Intro

**R2-1**. Line 75-76 “The findings of these studies suggest the importance of examining the impact of visual information on postural control” – suggested phrasing.

Response to [R2-1]: Thank you for your good suggestion. We revised the manuscript using your suggested phrasing (Please see lines 74-76).

**R2-2**. Line 81, 84 – remove “the” before postural instability

Response to [R2-2]: Thank you for your comments. “The” was removed, as your suggested (Please see lines 82 and 85).

**R2-3**. Line 87 – should this be “25% more body sway” instead of “25% of body sway”?

Response to [R2-3]: Thank you for your detailed comments. We revised the text from “25% of body sway” to “25% more body sway” according to your advice (Please see line 88).

Results:

**R2-4**. The postural instability index – appears to be a scalar (metric free) measure – is that correct? If it doesn’t have a metric – then you might consider providing a range of values so that the reader has a point of reference.

Response to [R2-4]: Thank you for your detailed comments. As you pointed out, the postural instability index is a scalar (metric free) measure, so the range of values was added as a point of reference for the reader as follows. (Please see lines 138-140).

“The postural instability index also increases with age as a result of degraded postural control (11.69±2.21 in normal young subjects and 24.84±6.07 in older subjects over 65 years old) [26].”

**R2-5**. Unless the journal requires exact values for reporting significance just use P < .05 or P > .05 as that was your criterion. Exact p values often lead people to think of an effect as more or less significant). If you wish to convey the practical significance – you can report effect sizes.

Response to [R2-5]: Thank you for your detailed comments. Based on your advice, we revised the p-values in Results section of main text as p < 0.05 or p > 0.05, but the p-values in Tables do not change to show exact effect sizes for reader (Please see the lines 203, 206, 207, 210, 214, 231, 234, and 237)

 < End of the response>
